# Predictive fatigue-aware human-robot collaboration: a real-time, open-source ROS 2 instantiation of a three-module human-centric digital twin framework on a standard cobot

**DOI:** 10.3389/frobt.2026.1897261

**Published:** 2026-08-04

**Authors:** Ranidu P. Goonetilleke, Azfar Khalid

**Affiliations:** Digital Innovation Research Group, Department of Engineering, School of Science and Technology, Nottingham Trent University, Nottingham, United Kingdom

**Keywords:** gesture recognition, human-centric digital twin, human-robot collaboration, Industry 5.0, intention prediction, operator fatigue, ROS 2, TEPR

## Abstract

Industry 5.0 cobotics calls for collaborative robots that adapt to the operator’s physical and cognitive state in real time. Most current industrial deployments remain reactive, responding only after explicit commands and ignoring the operator’s psychophysiological condition. The three-module Human-Centric Digital Twin (HCDT) framework establishes the upstream perception-and-reasoning architecture for such systems using Vision-Language Models, and identifies closed-loop feedback and physical-assistance mechanisms as the natural next layer in the framework’s development. Building on that foundation, this paper presents a complementary, real-time instantiation specialised for the resource-constrained, vision-plus-biosignal setting typical of ergonomics-and-safety HRC, deployed on a standard collaborative robot (Universal Robots UR3 with a Robotiq 2F-85 gripper) and extended with closed-loop operator-state adaptation. The Perception module pairs MediaPipe hand-landmark tracking and a Random Forest gesture classifier with Tobii Pro Glasses 3 pupillometry. The Reasoning module combines a ten-state finite-state machine, a weighted composite fatigue score (blink rate, blink duration, PERCLOS, hand-jerk) banded into FRESH, MILD, MODERATE and SEVERE, a saccade-gated commitment fixation, and a Task-Evoked Pupillary Response (TEPR) detector for acute-stress soft-emergency-stop. The Motion module provides a closed-form analytical inverse-kinematics solver, workspace clamping and a slew-rate-limited joint-velocity controller. Velocity-based linear extrapolation of hand position over a 0.5 s horizon enables anticipatory robot pre-positioning. The system is presented as a single-operator feasibility study rather than a generalisation claim. On a leak-free split-then-augment evaluation (520 raw test samples not seen by the training fold in any orientation), the Random Forest classifier reaches 99.62% accuracy with both errors falling in the Okay-Neutral confusion pair that the geometric override layer is designed to catch; a comparison against MLP and KNN baselines on the same split shows all three classifiers within the real-time control budget. Saccade-gated early-commit is designed to recover the modelled 150–300 ms gaze-to-motion lead, and the TEPR Soft E-Stop operates on an independent 300–500 ms channel. End-to-end gesture-to-motion latency has a conservative worst-case ceiling of approximately 830 ms, formed by a camera-buffer term, the 10-frame debounce and one control tick, inside the 1-s interactive response-time limit adopted as the design target. Owing to a Tobii Pro Glasses 3 hardware fault, the physiological pathways (fatigue banding, TEPR soft-stop and gaze commitment) were verified through their downstream control responses to simulated triggers rather than validated on organic pupil data; organic, multi-participant validation is identified as future work.

## 1 Introduction

Industrial operators and collaborative robots (cobots) are increasingly expected to share workspaces in direct coordination rather than strict segregation (Ajoudani et al., 2018; Wang et al., 2019). Despite the maturity of cobot hardware, two recurring limitations restrict the fluency of such collaboration. First, existing interaction interfaces (teach pendants, pre-programmed sequences, force-torque response) are fundamentally reactive: the operator must issue a completed command before the robot acts, and usability assessments show that programming through such interfaces is tedious and time-consuming (Villani et al., 2018). Second, the operator’s physiological and cognitive state is not accounted for in the control loop, despite well-documented evidence that fatigue degrades motor control, reaction time and decision quality (Ahsberg et al., 1997).

**TABLE 1:** Module-by-module comparison between the Asad et al. (2025) HCDT framework and the present instantiation. The architectural skeleton is preserved; the realisations are deliberately specialised for real-time, CPU-only operation on a standard cobot.

Module	Asad et al. (2025)	This work
Perception	SMPL-X parametric body, GVHMR, UniK3D 3D pose, vision-language gaze cues	MediaPipe 21 hand landmarks (21-D wrist-relative), Tobii Pro glasses 3 (blink rate, blink duration, PERCLOS, pupil diameter, gaze)
Reasoning	VLM with prompt engineering, two-phase task-state and intent prediction over a directed-acyclic task graph	10-state fetch FSM (NONE → IDENTIFIED → HOMING_TO_FETCH → PRE_APPROACHING → APPROACHING → WAITING_GRASP → GRASPING → RETURNING_HOME → DELIVERING → WAITING_RECEIVE) plus an orthogonal soft-e-stop scaling layer; composite fatigue F ( Equation 1 ); TEPR threshold p(t) > $\bar{p}$ (t − 3 s)·1.15; saccade-gated commitment (≥400 ms in 2°)
Motion	CLoSD/MDM diffusion-based 3D motion synthesis from HumanML3D representation	Closed-form analytical IK (3 free joints + 1 constrained wrist pitch, 2 wrist axes locked); workspace clamping; fatigue-adaptive interpolation; slew-rate-limited forward-velocity controller
Compute target	GPU-class hardware, multi-stage VLM and diffusion inference	Standard Intel i7 CPU, no GPU required; deterministic 20 Hz control loop
Feedback layer	Designated as future work	Closed loop: graded velocity scaling on chronic fatigue, OKAY-confirmation gate at MODERATE, full lockout at SEVERE, TEPR soft-e-stop on acute stress

This paper addresses both limitations in a single implementation. Predictive intention recognition extrapolates the operator’s hand trajectory from MediaPipe landmarks, allowing the robot to begin pre-positioning before a gesture is complete and recovering up to 0.5 s of effective latency. Continuous fatigue monitoring fuses four physiological indicators (blink rate, blink duration, percentage of eye closure or PERCLOS, and pupil diameter) acquired via the Tobii Pro Glasses 3 wearable eye tracker. Robot behaviour is dynamically modulated across four fatigue bands, from full-speed autonomy through OKAY-confirmation gating to a safety lockout.

Recent Human-Centric Digital Twin (HCDT) frameworks have established the upstream perception-and-reasoning architecture for such systems using Vision-Language Models, and have identified closed-loop feedback and physical-assistance mechanisms as the natural next layer (see Section 2.7). Building on that foundation, the pipeline described here instantiates the closed-loop HCDT for the resource-constrained, vision-plus-biosignal setting typical of ergonomics-and-safety HRC, by routing vision-based intent and pupillometric fatigue into the robot control law on commodity hardware, compatible with the small-and-medium-enterprise (SME) deployment envelope of contemporary HCDT proposals. Closely related, Escobar-Naranjo et al. (2026), in the same Research Topic, propose a methodological framework and experimental protocol for proactive HRC with multimodal intention prediction and adaptive control on a separate cobot platform; the present paper differs in delivering a closed-loop implemented system with measured timing, contact-free pupillometry-driven fatigue detection and a Task-Evoked Pupillary Response (TEPR) soft emergency stop, with a fuller comparison in Section 5.2.

The remainder of this paper is organised as follows. Section 2 surveys related work in human-robot collaboration, wearable and vision-based perception, anticipatory motion control, fatigue monitoring, collaborative safety standards and Human-Centric Digital Twins. Section 3 details the materials and methods, covering the gesture-recognition pipeline, velocity-based intention prediction, fatigue monitoring, TEPR-based acute-stress detection, the robot-control finite-state machine and motion controller, the visualisation layer and the hardware and software environment. Section 4 reports experimental results for classification accuracy, intention prediction, fatigue adaptation, state-machine behaviour and real-time performance. Section 5 discusses the significance of the findings, situates them against the existing HCDT literature and relates the system to collaborative-safety standards. Section 6 lists the limitations of the present study and outlines future work. Section 7 concludes.

## 2 Related work

### 2.1 Human-robot collaboration in Industry 4.0 and 5.0

Human-robot collaboration spans a range from shared-space coexistence to fully cooperative task execution. Hentout et al. (2019) review HRC developments between 2008 and 2017 and identify a persistent gap between human dexterity and robotic precision; effective systems must bridge this gap through intuitive interfaces that minimise operator cognitive burden. Wang et al. (2019) extend this view with the concept of symbiotic HRC, in which the robot holds explicit awareness of task state and of the operator’s intentions, capabilities and condition. The two perspectives converge on a requirement not met in combination by prior systems: proactive intent recognition paired with sensitivity to the operator’s current physiological state. More recent Industry 5.0 frameworks (Nasir et al., 2025) extend this view into a multi-level design-to-consumption framework within the Society 5.0 paradigm, sharpening the requirement that collaborative cobots adapt to operator condition in real time. The present work implements both layers.

### 2.2 Wearable and biosignal-based approaches

Intention recognition has been pursued through signals measured directly from the operator’s body. Bi et al. (2019) show that EMG classification of upper-limb motion can exceed 90% accuracy but requires electrode arrays whose performance is compromised by placement variability, skin impedance and sweat. Meng et al. (2016) demonstrate non-invasive EEG-based robot-arm control without surgical implantation, though their system relies on lengthy calibration, electromagnetic shielding and substantial operator training. A common limitation across biosignal approaches, including instrumented data gloves surveyed by Aditya et al. (2018), is that physical hardware must be attached to the operator, restricting natural dexterity and preventing rapid rotation between stations. Contact-free vision is therefore adopted in the present work as the primary intention modality.

### 2.3 Vision-based gesture recognition

Vision-based hand tracking has matured with learned landmark detectors. The MediaPipe Hands pipeline (Zhang et al., 2020) regresses 21 three-dimensional keypoints from a single RGB stream at over 30 frames per second on commodity hardware, without depth sensing. Downstream classification has been dominated by deep networks (Nuzzi et al., 2019), which deliver high accuracy but carry training-data, compute and latency costs that conflict with real-time robot control on commodity machines. Random Forests (Breiman, 2001) trade peak accuracy for bounded inference cost, interpretable decision boundaries and low sample requirements, properties better aligned with safety-critical deployment. A residual problem not resolved by the classifier alone is inter-gesture ambiguity under viewpoint variation, which is addressed in the present work through geometric validation.

### 2.4 Trajectory prediction and anticipatory control

Short-horizon human motion is sufficiently deterministic to be exploited for anticipatory robot control. Mainprice and Berenson (2013) estimate human goals using Gaussian Mixture Models so that robot motion planning can begin before the human reach is complete. Koppula and Saxena (2016) pursue the same objective through object-affordance-based activity anticipation, achieving horizons of several seconds by fusing scene context with observed motion. Both approaches require either learned motion models or semantically annotated scene graphs, which raise data-collection costs and do not translate trivially across unconstrained tasks; Unhelkar et al. (2018) nonetheless report 20%–30% cycle-time reductions from anticipatory behaviour. The present work adopts a lighter-weight linear extrapolation of hand velocity, trading predictive horizon for near-zero compute cost and trajectory-agnostic robustness.

### 2.5 Fatigue detection and operator monitoring

Operator fatigue is a documented safety concern: the UK Health and Safety Executive (HSE, 2021) attributes approximately 20% of workplace accidents to impaired alertness. Ji et al. (2004) establish PERCLOS as a reliable drowsiness indicator, while Soukupová and Čech (2016) introduce the Eye Aspect Ratio as an efficient blink-detection metric from which blink duration, shown by Benedetto et al. (2011) to increase markedly under fatigue, can be derived. Pupil diameter is identified by Marquart et al. (2015) as a complementary workload signal, and wearable eye trackers such as the Tobii Pro Glasses 3 (Tobii Pro, 2022) make both signals accessible during free movement. Outside the driving domain, Ahsberg et al. (1997) characterise the structure of occupational fatigue across industrial tasks, supporting the transfer of the eye-based and motor metrics described above into manufacturing settings, although per-operator threshold calibration remains a recognised requirement. Carissoli et al. (2024) note that few HRC systems dynamically adapt robot behaviour to detected fatigue. This gap is directly addressed in the present work through a composite fatigue score coupled to the robot control law.

### 2.6 Safety standards in collaborative robotics

Collaborative-robot safety is governed internationally by ISO 10218-1:2025 (ISO, 2025) and ISO 15066:2016 (ISO, 2016), which define safety-rated monitored stop, hand guiding, speed-and-separation monitoring, and power-and-force limiting as permissible collaborative modes. Robla-Gómez et al. (2017) review industrial implementations of these standards and observe that effective safety systems depend on layered perception accommodating both predictable and unpredictable human behaviour. Both treat operator capability as constant; fatigue-induced changes in reaction time and control precision sit outside the current scope of the standards. The mechanisms developed in the present work are research-layer safety behaviours, not certified safety functions: the gesture STOP and the software workspace clamping are inspired by the intent of safety-rated monitored stop and speed-and-separation monitoring respectively, but they run on non-safety-rated sensing and control and are not equivalent to the certified functions the standards define. They are best understood as a research-layer extension that additionally modulates robot behaviour against the operator’s measured state.

### 2.7 Human-centric digital twins

Asad et al. (2023) and Asad et al. (2025) provide the principal review and current framework for HCDTs. The review establishes the symbiotic perception–cognition–interaction loop around the human worker, and the framework extends this with three modules (Perception and Scene Representation, AI-driven Reasoning and Prediction, and Motion Generation) that use SMPL-X parametric body models, Vision-Language Models with prompt engineering, and CLoSD/MDM diffusion-based motion synthesis. The framework targets rich semantic understanding in unconstrained environments and scopes the closed-loop feedback and physical-assistance layers as the natural next stage of development. The present paper preserves the same three-module skeleton and selects deterministic, CPU-only module realisations appropriate to a real-time HRC deployment on a single cobot, supplying the closed-loop feedback layer that the framework scopes as outstanding (Table 1).

Building on Asad et al. (2025), a wave of more recent contributions has refined the picture. Villani et al. (2025) propose an Operator Digital Twin and Machine Digital Twin (ODT/MDT) ecosystem for Industry 5.0 with biometric shadowing of the operator’s physical, emotional and cognitive states. Abellán et al. (2026) provide a PRISMA-style review that identifies six dimensions of Human Digital Twins (physical, cognitive, social, emotional, cultural and ethical) and notes that earlier HCDT literature has tended to treat the human as an agent in the environment rather than as the modelled subject. Lauer-Schmaltz et al. (2024) distinguish shallow from deep Human Digital Twins in their ETHICA methodology, reserving the deep category for systems that close the loop on user state. Within the same research group as the present work, Khan et al. (2023) examine Vicon-based human motion analysis for HCDT applications; the present paper extends that programme towards closed-loop, real-time operator-state-adaptive control on commodity hardware. A recurring observation across these contributions is that closed-loop physiological feedback from operator to robot behaviour remains rare in practice, a gap directly addressed by the present work.

## 3 Materials and methods

The system is implemented entirely in ROS 2 Humble (Macenski et al., 2022) on the Universal Robots UR3 with a Robotiq 2F-85 gripper. Perception is contactless; no hand-mounted instrumentation is worn. The primary contributions are: (i) a real-time MediaPipe-plus-Random-Forest gesture pipeline augmented with geometric validation; (ii) velocity-extrapolation intention prediction visualised as a ghost target; (iii) a composite fatigue score fusing eye-tracking and hand-jerk metrics; (iv) an adaptive control architecture coupling robot behaviour to operator state; and (v) a complete open-source ROS 2 implementation. The hypothesis tested is that a closed-loop HCDT instantiation on commodity, CPU-only hardware can meet sub-second perception-to-actuation latency while modulating robot behaviour against measured operator fatigue on a single standard cobot.

**FIGURE 1:**
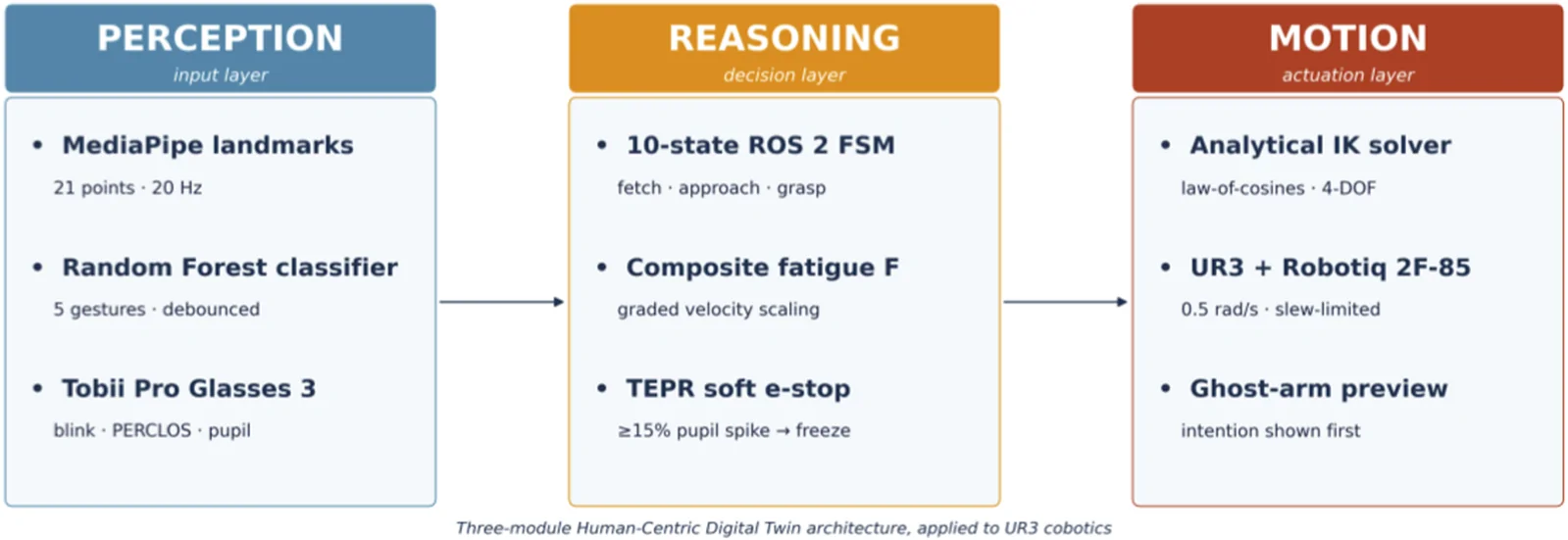
Three-module HCDT architecture: perception (camera + eye-tracker), Reasoning (FSM + composite fatigue + TEPR), Motion (analytical IK + slew-limited velocity).

The system architecture follows a modular publish-subscribe design. The system is organised as three functionally distinct modules, each realised as one or more ROS 2 nodes: a Perception Module (camera_node, tobii_node) that converts raw RGB imagery and the wearable eye-tracker stream into structured signals; a Reasoning Module (camera_node downstream stages and fatigue_monitor) that fuses perception outputs to estimate the current gesture, short-horizon hand target, cognitive-commitment state via saccade classification, acute stress via TEPR, and composite fatigue; and a Motion Generation Module (robot_controller) that consumes these estimates and synthesises safety-bounded motion through a fatigue-adaptive state machine coupled to a real-time analytical IK solver and slew-limited joint-velocity controller. This decomposition, illustrated in Figure 1, allows individual modules to be upgraded without disrupting the pipeline.

### 3.1 Gesture recognition pipeline

The gesture-recognition pipeline operates in three stages: hand detection and landmark extraction, feature computation, and classification with geometric validation. Hand detection and landmark regression are performed by the MediaPipe Hands model (Zhang et al., 2020), configured for single-hand tracking with a detection-confidence threshold of 0.7 and a tracking-confidence threshold of 0.5. The model produces 21 three-dimensional landmarks per detected hand, including the wrist, each finger’s metacarpophalangeal, proximal interphalangeal and distal interphalangeal joints, and fingertips.

Raw landmark coordinates are normalised relative to the wrist position (landmark 0) to achieve translation invariance. For each frame, the wrist position is subtracted from all 21 landmarks, producing a 63-dimensional feature vector (21 landmarks × 3 coordinates) that represents hand pose independent of absolute position within the camera frame. This normalisation ensures that the classifier responds to hand configuration rather than hand location. Data augmentation is performed during training through horizontal mirroring of the landmark data (negating the x-coordinate), effectively doubling the training set and enabling ambidextrous recognition without requiring separate training for left- and right-handed operators.

A Random Forest classifier (Breiman, 2001) with 100 estimators is trained on the normalised landmark features to discriminate five gesture classes: POINT (index finger extended, directing the robot to a target location), STOP (open palm, immediately halting robot motion), OKAY (thumb-index circle, confirming pending actions or unfreezing the robot), GRASP (closed fist, commanding gripper closure), and NEUTRAL (relaxed hand, no active command). A sixth gesture, TOGGLE (the shaka hand sign), is recognised by a deterministic finger-extension rule rather than by the Random Forest: it is applied in the geometric-override layer and overrides the Random-Forest output when the thumb-and-pinky-extended pattern is present. More generally, to resolve inter-gesture ambiguity under viewpoint variation, this override layer applies two kinds of scale-invariant finger-extension rules: correction rules that downgrade likely misclassifications (for example, forcing POINT when the index is clearly the dominant extended finger, or GRASP when no finger is extended) and pose rules that assign gesture identity directly from finger geometry (for example, a fist to GRASP and a thumbs-up to OKAY); the full rule set is provided in the released code. A consecutive-frame debounce then emits a new gesture label only after it has appeared in ten consecutive frames, with the STOP gesture receiving a faster two-frame debounce to preserve safety responsiveness, preventing single-frame misclassifications from triggering unintended robot actions.

Five layered noise-suppression mechanisms sit between the raw camera frame and any committed gesture-driven robot motion. The first is the MediaPipe model’s own confidence floor: a detection-confidence threshold of 0.7 and a tracking-confidence threshold of 0.5, which together reject frames in which the hand detector itself is uncertain. The second is the velocity gate: when the fingertip speed of the index and middle fingers exceeds 1.8 normalised image-coordinate units per second, landmark republication is frozen and the last stable frame is re-published, so the classifier sees a settled hand shape rather than a transient morph; the gate releases with hysteresis at 0.6 units per second. The third is the Random Forest probability gate: when the classifier’s top-class posterior falls below 0.7, the previously committed gesture is held rather than overwritten with an uncertain prediction. The fourth is the geometric-validation override layer described above, which rewrites the classifier’s output when scale-invariant finger geometry contradicts it. The fifth is the consecutive-frame debounce: a new gesture must appear in 10 consecutive frames before it is committed, with the STOP gesture receiving a faster two-frame debounce to preserve safety-command responsiveness. Each layer addresses a different failure mode, and removing any one degrades either responsiveness or false-trigger rate measurably during deployment.

### 3.2 Intention prediction through velocity extrapolation

Beyond recognising the current gesture, the system predicts the operator’s future target by tracking hand velocity over time. The wrist landmark position is recorded at each frame, and a three-frame interval is used for finite-difference velocity estimation, yielding smoother estimates than consecutive-frame differencing. The predicted future position is obtained through linear extrapolation of this velocity over a 0.5 s horizon, clamped to the valid coordinate range to prevent artefacts. Prediction confidence is derived from the instantaneous speed, reflecting the principle that faster, more deliberate motions yield more reliable linear extrapolation.

The MediaPipe kinematic predictor cannot on its own distinguish committed motion from exploratory drift. The Tobii Pro Glasses 3 gaze stream is therefore fused into the prediction pipeline as a cognitive authorisation gate: a commitment fixation is declared when gaze remains within a 2° angular window for ≥ 400 ms (Yarbus, 1967; Hayhoe and Ballard, 2005), after which the 0.8 cap on the prediction confidence is lifted and pre-positioning is authorised. The mechanism is designed to exploit the gaze-precedes-reach relationship, which models a 150–300 ms lead between gaze commitment and hand-motion onset; because the eye-tracker ran in simulation during the trials (Section 4.3), this lead is a modelled value rather than a measured one, and live measurement is identified as future work (Section 6).

### 3.3 Fatigue monitoring

The fatigue-monitoring subsystem fuses data from the Tobii Pro Glasses 3 wearable eye tracker with hand-movement metrics derived from the camera-based tracking system. Four metrics contribute to a weighted composite fatigue score.

Blink rate is the number of blinks within a trailing 60-s window, linearly mapped to a score in [0, 1] between a rested baseline and a fatigued ceiling (nominally 12 and 28 blinks per minute; the exact values are configurable parameters informed by the fatigue literature, e.g., Benedetto et al., 2011). Blink duration is measured as the interval between eye openness falling below 0.5 and returning above 0.5; the mean over the 10 most recent blinks (from a rolling 30-blink buffer) is mapped to a [0, 1] score between nominal baseline and fatigued values (0.12 and 0.38 s; Soukupová and Čech, 2016). PERCLOS is computed as the proportion of eye-openness samples below the 0.5 threshold within a trailing 60-s window and mapped to a [0, 1] score against a nominal threshold (0.08; Ji et al., 2004). Hand-movement smoothness is quantified through a hand-jerk metric, computed as the mean absolute difference between consecutive hand-speed measurements; fatigued operators exhibit increased tremor and less smooth trajectories, resulting in elevated jerk values, which are normalised to a [0, 1] score.

The four metric scores are combined through weighted averaging:

$$\begin{array}{ll} & F=0.30\times S\_\mathrm{blink}\_\mathrm{rate}+0.25\times S\_\mathrm{blink}\_\mathrm{duration}+0.30\\ & \;\times S\_\mathrm{PERCLOS}+0.15\times S\_\mathrm{hand}\_\mathrm{jerk} \end{array}$$ (1)

where F is the composite fatigue score and the four sub-scores are the normalised values defined above. The weights were set nominally to favour the more reliable eye-based metrics (Marquart et al., 2015) and were held fixed for this work; the band thresholds (0.30, 0.60, 0.80) follow the same convention. Per-operator calibration of both the weights and the band thresholds from a short baseline-recording phase at session start is identified as future work in Section 6. The composite score F ranges from 0.0 (fully alert) to 1.0 (severely fatigued) and is banded into four states: FRESH (F < 0.30), MILD (0.30 ≤ F < 0.60), MODERATE (0.60 ≤ F < 0.80) and SEVERE (F ≥ 0.80). The four bands map onto four robot-behaviour bands, described in Section 3.5.

### 3.4 Acute stress detection via TEPR

Beyond chronic fatigue monitoring, the system performs acute-stress detection through pupillometry. Sudden high-velocity pupil dilation is a validated autonomic biomarker of sympathetic arousal with a 300–500 ms detection latency, faster than conscious reach-to-emergency-stop reaction time of approximately 400–600 ms (Beatty, 1982; Laeng et al., 2012; Woodworth, 1938). The Tobii Pro Glasses 3 stream is monitored against a rolling 3-s baseline to isolate stress-related dilations from the Pupillary Light Reflex. The 15% spike threshold and 3-s baseline window are nominal values informed by the pupillometry literature (Beatty, 1982; Laeng et al., 2012); per-operator calibration is treated as future work alongside the chronic-fatigue thresholds of Section 3.3. A dilation exceeding 15% within a 0.5 s window triggers a Soft E-Stop that scales both the velocity ceiling and slew cap by 0.1, producing a tenfold deceleration that artificially expands the operator’s reaction window. In the current evaluation the trigger is simulated via Numpad 5 using Wizard-of-Oz methodology (Dahlbäck et al., 1993), for the reasons described in Section 4.4.

### 3.5 Robot control and state machine

**FIGURE 2:**
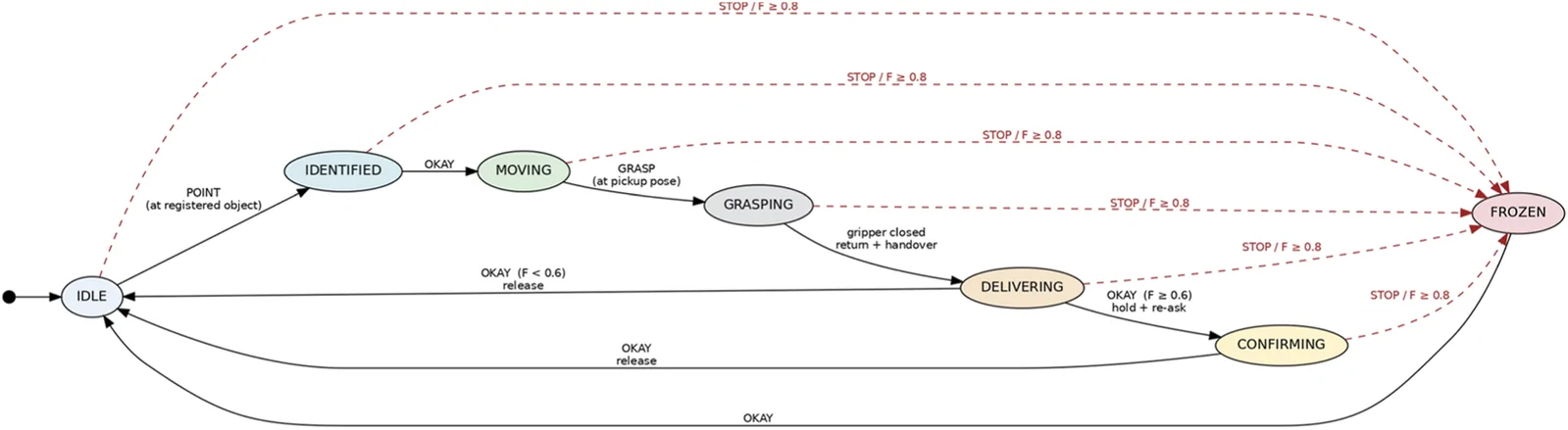
Fetch finite-state machine. The diagram shows a collapsed seven-node view of the underlying ten-state controller, grouping the four spatial transit states (HOMING_TO_FETCH, PRE_APPROACHING, APPROACHING, RETURNING_HOME) under a single MOVING node for readability; the soft-e-stop and SEVERE-fatigue branches (dashed red) latch the current state into a frozen register, from which an OKAY gesture in the absence of severe fatigue resumes operation.

The robot controller implements a fetch finite-state machine with ten internal states: NONE (idle, awaiting gesture input), IDENTIFIED (a target has been pointed at and committed), HOMING_TO_FETCH (moving to a safe pre-grasp pose above the target), PRE_APPROACHING (a target-conditional intermediate waypoint, entered only for the Red Cube, whose position nearest the workspace edge requires an alignment step to avoid a singularity-adjacent direct approach; the other targets transition straight to APPROACHING), APPROACHING (descending toward grasp pose), WAITING_GRASP (positioned at pickup, awaiting an explicit GRASP gesture), GRASPING (gripper is closing), RETURNING_HOME (transporting the held object to the delivery zone), DELIVERING (descending to release), and WAITING_RECEIVE (positioned at handover, awaiting an OKAY gesture to release). The complete transition graph is summarised in Figure 2 and enumerated in the released codebase. Two safety mechanisms operate orthogonally to the fetch FSM: a STOP gesture or a SEVERE fatigue reading (F ≥ 0.8) parks the current fetch_state in a frozen-state register and zeroes the commanded velocity, with resumption gated on an OKAY gesture in the absence of severe fatigue; and the TEPR soft-e-stop applies a multiplicative estop_scale of 0.1 to the velocity ceiling and slew cap for the duration of a 3-s cooldown window. Confirmation gating at MODERATE fatigue (F ≥ 0.6) is implemented inside WAITING_GRASP and WAITING_RECEIVE rather than as a separate state.

Continuous torch-mode tracking (a sustained POINT driving the end-effector to follow the fingertip directly) is implemented as a separate FSM and is not shown in Figure 2. A MODERATE fatigue reading pauses the tracker until a further OKAY re-confirms alertness. Low-level motion generation uses a closed-form analytical inverse-kinematics solver: three free joint angles (shoulder pan, shoulder lift, elbow) are computed to reach the (x, y, z) target, and the wrist pitch is then derived as wrist_1 = −π/2 − q_2_ − q_3_ to maintain a vertical end-effector, where q_2_ and q_3_ are the shoulder-lift and elbow angles. The remaining two wrist axes (wrist_2 = −π/2, wrist_3 = 0) are locked at fixed values for the top-down grasping configuration. This reduces the general 6-DOF inverse problem to a closed-form solution plus an orientation constraint, avoiding the iterative numerical search that a general 6-DOF IK would require. The solver is combined with workspace clamping, a fatigue-adaptive interpolation factor, and a slew-rate-limited forward-velocity controller. Two distinct fatigue-scaling layers modulate the controller. The torch-mode stick-speed multiplier scales the operator-driven velocity command by 1.00, 0.70, 0.40 and 0.15 across FRESH, MILD, MODERATE and SEVERE respectively, capping the maximum commanded velocity. The slew-rate multiplier independently scales the per-tick rate of change of the commanded velocity by 1.000, 0.625, 0.375 and 0.000, so that at SEVERE the commanded velocity is held constant rather than allowed to ramp upward. Acting together, the two layers produce a graded slowdown at MILD and MODERATE, and a velocity hold at SEVERE that complements the FSM lockout.

The deployed system uses forward velocity control with slew-rate limiting rather than impedance or admittance control. Collaborative safety is provided by four complementary mechanisms: gesture-driven discrete stops (STOP gesture and the FROZEN state), operator-state-adaptive velocity scaling across the four fatigue bands, geometric envelopes (workspace clamping and hazard-zone denial), and the UR3 firmware’s built-in joint-torque collision detection underneath the ROS 2 layer. Direct-contact collaboration via Cartesian impedance control would be a complementary extension for force-modulated tasks such as surface polishing, deburring or hand-guided teaching, and is identified as future work in Section 6.

### 3.6 Visualisation

**FIGURE 3:**
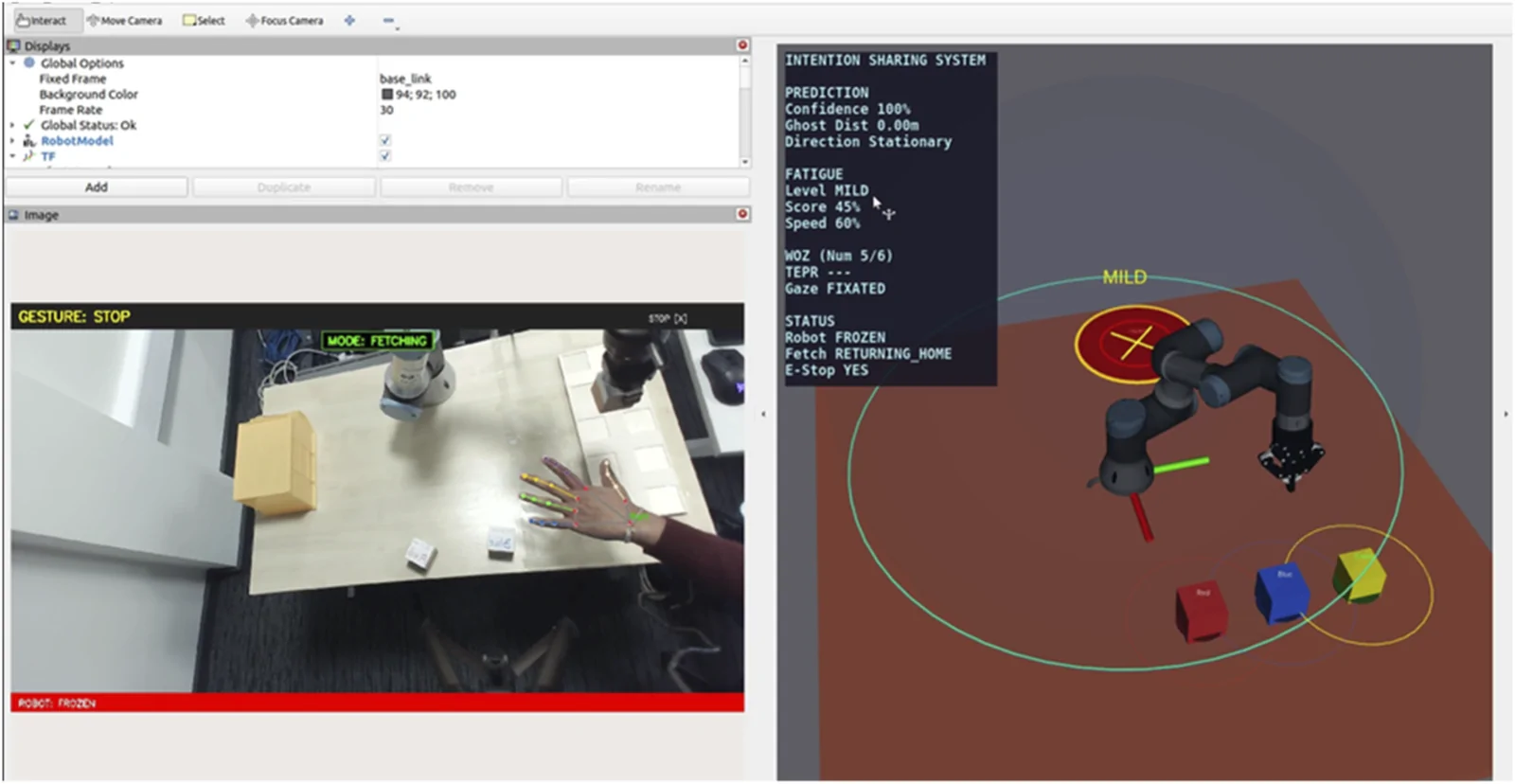
RViz HUD during a live STOP-gesture demonstration: gesture text “STOP” recognised with MediaPipe landmarks visible on the camera feed; right pane shows the robot frozen with ghost-arm preview suppressed.

**FIGURE 4:**
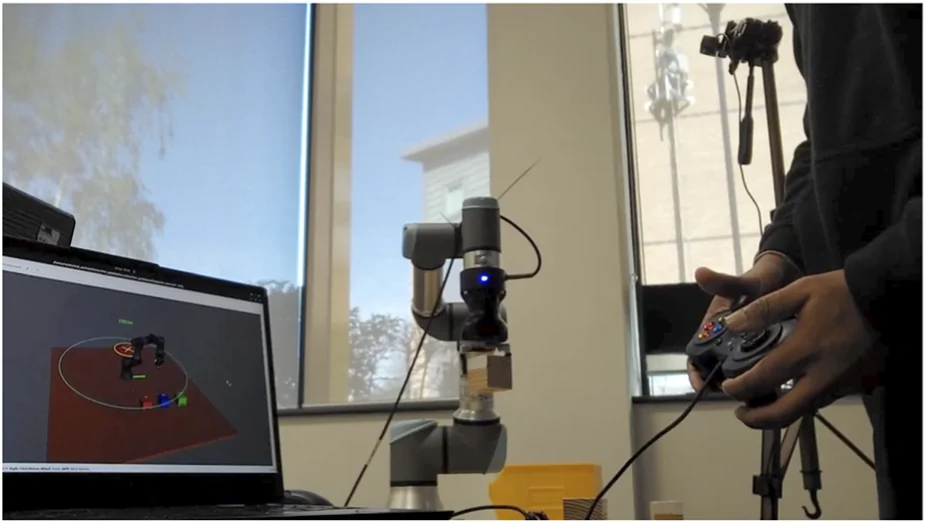
Live UR3 + Robotiq 2F-85 cell, with overhead RGB webcam mounted on the tripod and a gamepad fallback in the foreground. The three coloured fetch cubes and the hazard-zone marker (yellow bin) are visible on the table.

**FIGURE 5:**
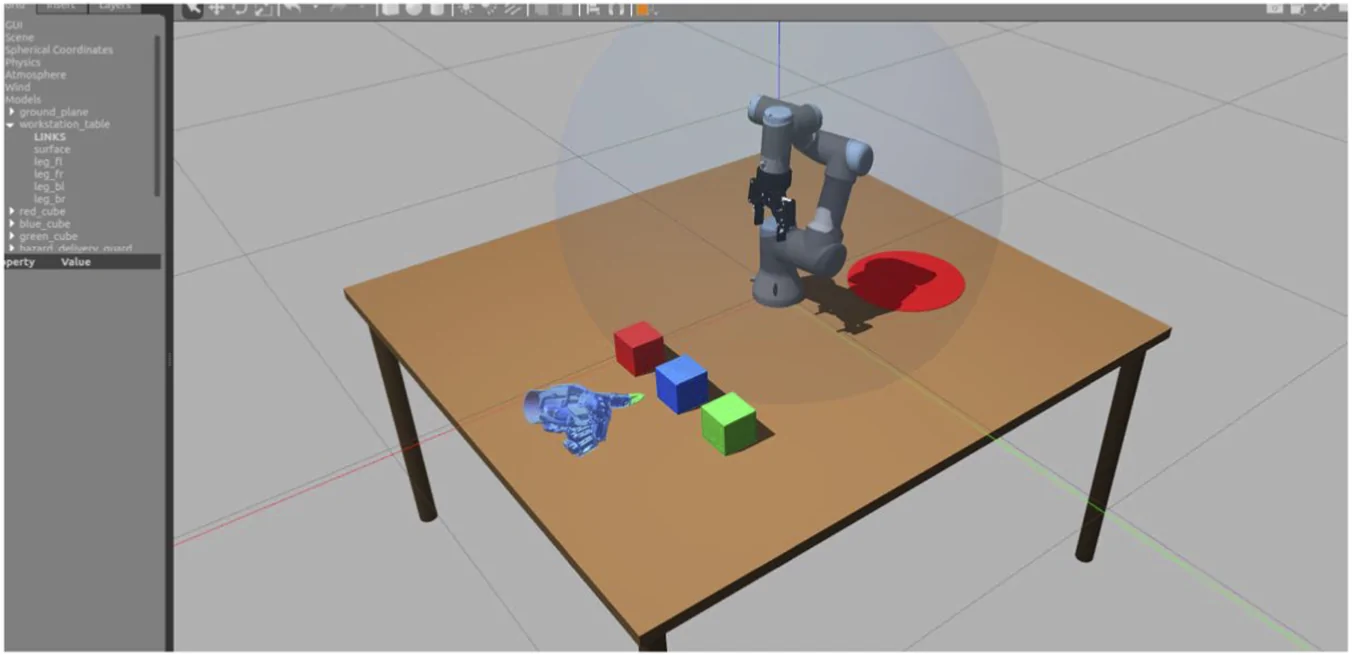
Gazebo Classic 11 digital twin of the cell, running the same ROS 2 Humble stack as the physical hardware. The full FSM, IK solver, fatigue-speed coupling and slew-limited velocity controller were iterated in simulation before deployment to the physical UR3.

The system provides full visual feedback through RViz2, including a red arrow for the detected pointing direction; green and red spheres at the clamped and raw target positions (connected by a yellow correction line when clamping is active); a blue sphere for the workspace reach boundary; a brown plane for the table surface; a purple ghost sphere at the predicted future position, scaled and labelled by confidence; and a floating-text display of the current robot state. The camera feed is augmented with an overlay showing the current gesture, the corresponding robot action, and a highlighted fingertip marker when pointing. Figures 3–5 illustrate the system in operation.

### 3.7 Hardware, software environment and materials

**FIGURE 6:**
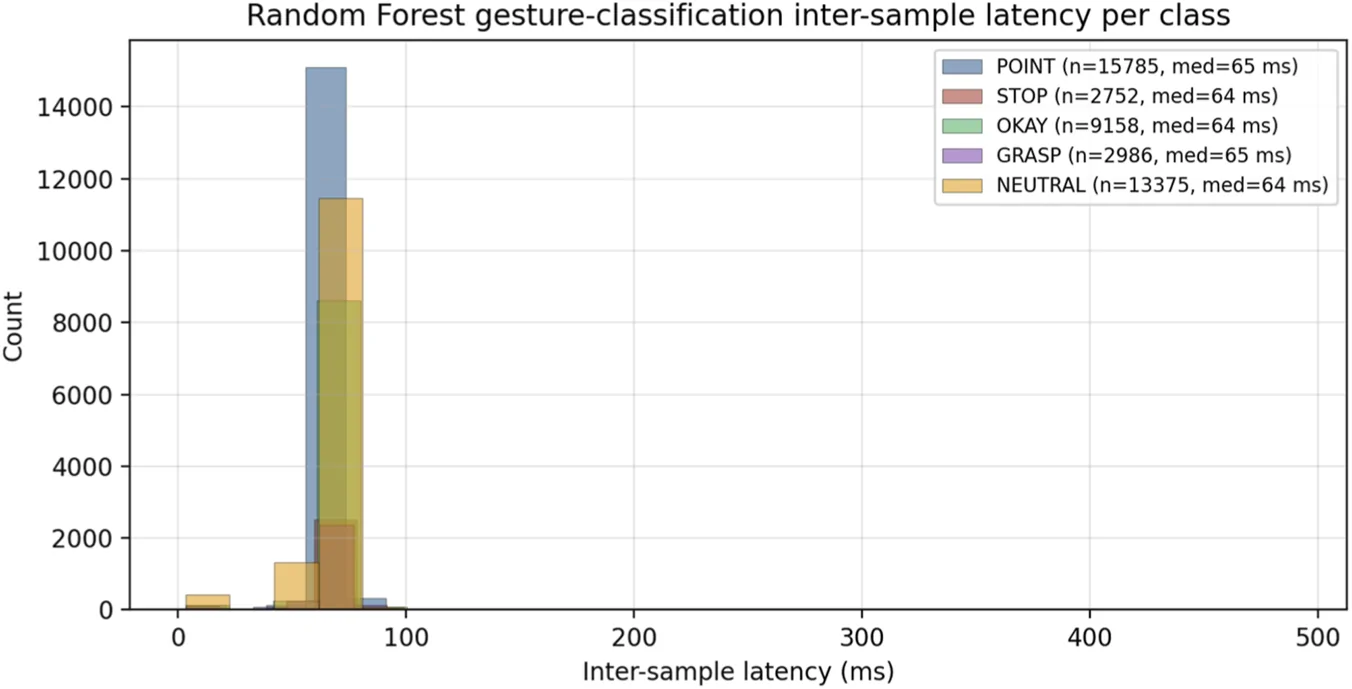
Random forest gesture-classification inter-sample latency per class from latency_log.csv. Median latency is 64–65 ms across the five Random Forest classes (POINT, STOP, OKAY, GRASP, NEUTRAL) with 95th-percentile values of 69–77 ms, consistent with the approximately 15 Hz MediaPipe inference rate on 1080p input. The downstream forward-velocity control loop runs at 20 Hz and samples the latest published gesture. The deterministic TOGGLE detector is excluded because it is a deterministic override rather than a Random-Forest class.

The robotic platform comprises a Universal Robots UR3 collaborative manipulator with six degrees of freedom and a maximum reach of 0.5 m, equipped with a Robotiq 2F-85 adaptive gripper providing an 85 mm stroke with position control. The vision system uses an overhead-mounted Logitubo 920 USB webcam (T/N 920H4, P/N USBL-232H4) capturing 1920 × 1080 MJPEG frames; the perception node schedules MediaPipe Hands at a 30 Hz timer but achieves an effective rate of approximately 15 Hz on the deployment laptop CPU, the limiting factor being full-HD landmark inference (Figure 6). The operator interacts single-handed; the detector permits two hands but the pipeline uses the operating hand. Eye tracking is performed by the Tobii Pro Glasses 3, whose 50 Hz gaze stream is consumed by the pipeline at 10 Hz via WiFi connection. The system runs on Ubuntu 22.04 LTS with ROS 2 Humble Hawksbill; the inter-node topics, message types and rates are summarised in Table 2. Key software dependencies include MediaPipe 0.10.x for hand-landmark detection, scikit-learn 1.x for Random-Forest classification, OpenCV 4.x for image processing, g3pylib for Tobii Pro Glasses 3 communication, tf2 for coordinate-frame transformations, and Gazebo Classic 11 with the Open Dynamics Engine physics solver for digital-twin simulation. The IFRA LinkAttacher plugin (IFRA-Cranfield, 2023) simulates grasping by welding a fixed joint to wrist_3_link; the default Gazebo contact solver is unable to sustain stable grasps across a complete fetch cycle without this workaround, although the physical UR3 hardware does not require it. Figures 4, 5 above show the live and simulated cells respectively.

The full software stack was iterated against the Gazebo twin before each physical-hardware session, enabling unsafe parameter ranges (fatigue-band velocity scaling, slew-rate caps, IK joint limits) to be exercised without risk to operator or robot. This separation of software iteration from hardware availability proved load-bearing during sessions in which laboratory access was constrained, and underlies the choice to maintain the simulated and physical control pipelines as byte-identical ROS 2 stacks rather than parallel implementations. The simulator is used as a development and edge-case-testing environment for the deterministic control logic, not as a substitute for physical-hardware validation of contact dynamics or sensor noise, which remain the responsibility of the live trials reported in Section 4.

Three coordinate frames are defined, base_link at the robot base, camera_link for the RGB camera, and an implicit Tobii scene-camera frame, related by a static transform publisher. The non-orthogonal overhead view of the tripod camera is corrected by a 5-point homography calibration that maps image pixels to the base_link ground plane via cv2.findHomography (Hartley and Zisserman, 2004). The full calibration procedure, the projective mapping derivation and the runtime cv2.perspectiveTransform application are documented in the released repository.

Gesture training data were collected using a custom recording node that subscribes to the hand-landmark topic and writes timestamped landmark vectors to CSV files. The raw per-class counts in the released dataset are 649 (GRASP), 523 (POINT), 520 (STOP), 526 (OKAY) and 585 (NEUTRAL); during recording, the operator deliberately varied hand orientation, position within the frame, distance from the camera, and movement direction to prevent overfitting to specific hand poses. The classes are balanced down to the smallest count (520) to give 2,600 raw samples in total, and a stratified 80/20 split is then applied to that raw corpus (2,080 train, 520 test). Mirror-augmentation across the wrist’s vertical axis is applied only to the training fold after the split, supporting left- and right-handed operators while preserving an unaugmented test set in which no sample’s mirror twin appears in training. The resulting pipeline produces 4,160 training samples (2,080 raw plus 2,080 mirrored) and 520 leak-free test samples (104 per class). This split-then-augment ordering is a deliberate departure from the augment-then-split protocol originally used in early-development evaluations; an augment-then-split corpus places each test frame’s mirror twin in the training set, which collapses the generalisation signal. The leak-free pipeline reported here is the one shipped in the released repository, and it produces the classifier and accuracy figures reported in Section 4.1.

## 4 Results

All results in this section are obtained from a single-operator, within-session evaluation and are reported as a feasibility demonstration; they characterise the implemented system rather than its generalisation across operators or sessions. The section reports empirical results across five evaluation axes: gesture classification accuracy on the within-session test set; saccade-gated intention-prediction timing; the four-band fatigue discretisation and the TEPR-triggered soft-emergency-stop; the verified state-machine transitions; and real-time end-to-end latency.

### 4.1 Gesture classification performance

**FIGURE 7:**
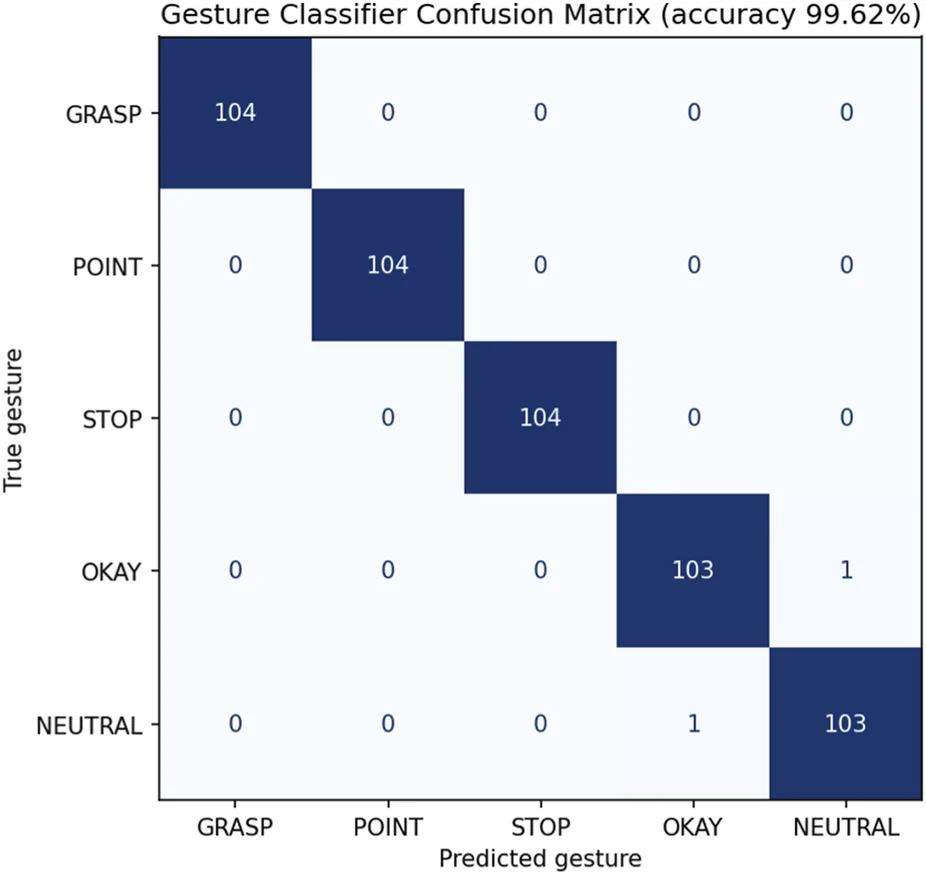
Gesture classifier confusion matrix on the leak-free test set (520 raw samples, 104 per class). Both Random Forest errors are Okay-Neutral confusions, which the geometric override layer of Section 3.1 catches at the system level via a scale-invariant thumb-extension check.

Three classifier families were evaluated on the leak-free split described in Section 3.7: an 80/20 stratified split of the 2,600 raw samples followed by mirror-augmentation of the training fold only (4,160 train, 520 raw test, 104 per class). On this split, the Random Forest classifier deployed in the system achieves 99.62% test accuracy (518/520 correct). Both errors fall in the Okay-Neutral confusion pair, which is the gesture pair distinguished primarily by thumb extension, a landmark axis known to be more MediaPipe-noisy than the four-finger curl. The confusion matrix is shown in Figure 7; per-class precision, recall and F1-score are given in Table 3.

**FIGURE 8:**
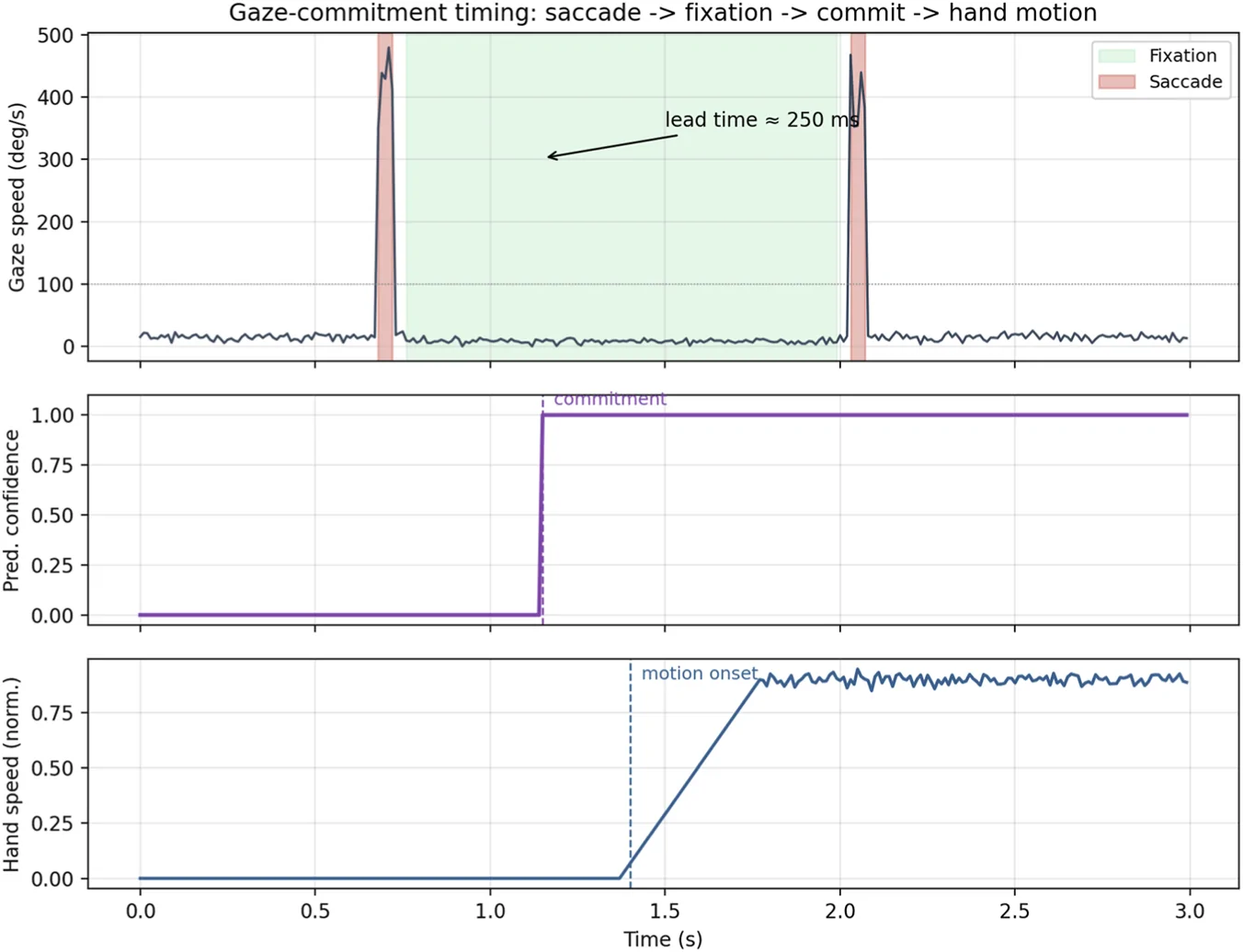
Illustrative (modelled) gaze-commitment timing diagram generated from a synthetic trace, not from logged trial data or the deployed confidence computation. Top panel: gaze angular velocity with saccade (red) and fixation (green) segments. Middle: the commitment gate, drawn as a binary signal that rises once gaze has dwelt 400 ms within the 2° window; in the deployed system commitment lifts the 0.8 cap on the speed-derived confidence (Section 3.2) rather than setting it to 1.0. Bottom: hand-motion onset following commitment by a modelled approximately 250 ms, within the 150–300 ms gaze-to-motion lead reported in the literature.

**TABLE 2:** ROS 2 topic inventory for the deployed pipeline. Reliable QoS with transient-local durability is used for state and fatigue topics so that late subscribers see the latched value; sensor and command streams use best-effort QoS.

Topic	Type	Publisher → subscribers	Rate
/camera/image_raw	sensor_msgs/Image	camera_node → MediaPipe stage	30 Hz
/hand/landmarks	Float32MultiArray (63-D)	camera_node → robot_controller, fatigue_monitor	30 Hz
/gesture/text	std_msgs/String	camera_node → robot_controller	20 Hz (smoothed)
/intention_marker	geometry_msgs/PointStamped	camera_node → robot_controller	20 Hz
/hand_velocity	geometry_msgs/Vector3	camera_node → robot_controller	20 Hz
/eye_tracking/raw	Float32MultiArray (8-D)	tobii_node → fatigue_monitor	10 Hz
/eye_tracking/gaze_point	geometry_msgs/PointStamped	tobii_node → robot_controller	10 Hz
/soft_estop	std_msgs/Bool	tobii_node → robot_controller	20 Hz
/gaze/committed	std_msgs/Bool	tobii_node → robot_controller	20 Hz
/fatigue/score	std_msgs/Float32	fatigue_monitor → robot_controller	5 Hz
/fatigue/level	std_msgs/String	fatigue_monitor → robot_controller	5 Hz (transient-local)
/robot/state	std_msgs/String	robot_controller → RViz, logger	5 Hz (transient-local)
/arm/velocity_controller/commands	std_msgs/Float64MultiArray (6-D)	robot_controller → UR3/Gazebo	20 Hz

**TABLE 3:** Per-class precision, recall, F1-score and support for the Random Forest classifier on the leak-free test set. The TOGGLE (shaka) gesture is excluded by construction because it is recognised by a deterministic finger-extension rule rather than the Random Forest.

Gesture	Precision	Recall	F1-score	Support
GRASP	1.00	1.00	1.00	104
POINT	1.00	1.00	1.00	104
STOP	1.00	1.00	1.00	104
OKAY	0.99	0.99	0.99	104
NEUTRAL	0.99	0.99	0.99	104
Overall	1.00	1.00	1.00	520

Alternative classifiers were evaluated on the same leak-free split to characterise the design space. A two-hidden-layer multilayer perceptron (MLP, hidden sizes 64 and 32) and a k-nearest-neighbours classifier (k = 5) both achieved zero test errors on the 520-sample test set; the difference between these and the Random Forest’s two Okay-Neutral confusions is at the edge of statistical noise at this test-set size and is not interpreted as a meaningful generalisation advantage. Table 4 summarises the comparison.

**TABLE 4:** Classifier comparison on the leak-free split. Train and inference times are from a representative run on the test machine (Intel i7 CPU); sub-millisecond inference is machine-specific and cache-sensitive. All three classifiers sit several orders of magnitude inside the 50 ms control-loop budget; KNN’s inference time grows with the training-set size because it computes distances against all 4,160 training samples at prediction time.

Classifier	Accuracy	Train time (s)	Inference (μs/sample)	Model size (KB)
Random forest (100 trees)	99.62%	≈1.0	≈10	1,338.0
MLP (64, 32)	100.00%	≈2.3	≈1–2	206.0
KNN (k = 5)	100.00%	≈0.0	≈90–175	2,080.7

The deployed system retains the Random Forest classifier despite the marginal accuracy gap to MLP and KNN, for three engineering reasons. First, the geometric override layer of Section 3.1 is designed against the Random Forest’s feature-importance output, which directly identifies thumb-extension and finger-curl axes as the most discriminative features for the five-class problem; both of the Random Forest’s test errors fall in exactly the Okay-Neutral confusion that the override layer is designed to catch via a scale-invariant thumb-extension check; quantifying the override layer’s effect on cross-session, cross-operator deployment is reserved for the multi-participant evaluation described in Section 6. The MLP’s weights are not interpretable enough to design or maintain equivalent geometric rules. Second, all three classifiers fit the real-time budget with substantial margin: at approximately 10 μs per sample, the Random Forest is three to four orders of magnitude inside the 50 ms control-loop period, so inference latency was not a deciding factor. Third, the Random Forest’s robustness to small-dataset training and absence of hyperparameter sensitivity make it appropriate for the deployment envelope (commodity CPU, no per-deployment retraining). For external comparison, prior vision-based HRC gesture systems report 91.3% (CNN, Nuzzi et al., 2019) and approximately 95% (data-glove, Aditya et al., 2018) accuracy on broadly comparable problems, neither of which incorporates predictive intention or fatigue adaptation.

### 4.2 Intention prediction

The velocity-based prediction system is characterised here through its construction and a *post hoc* audit of the released prediction log (gaze_log.csv). By construction, the ghost target leads the mapped hand position by the 0.5 s extrapolation horizon multiplied by the current hand velocity, so during deliberate sweeps it appears ahead of the hand in its direction of motion; because the predicted-endpoint stream was not written to disk, no measured lead distance or endpoint error is reported (Table 5, Part B). The displayed prediction confidence is a deliberateness indicator, not an accuracy measure: during free hand-following it equals the normalised hand speed by construction, rising during fast deliberate motion and decaying as the hand slows, while during an active fetch it is pinned at 1.0 and capped at 0.8 whenever the simulated gaze stream was not in a committed fixation. The prediction log records one row per hand-velocity event outside torch mode and pools webcam-sourced and virtual-operator-sourced sessions; excluding its first five sessions, which predate a change to the confidence divisor, the pipeline was engaged (non-zero confidence) in 35.3% of the 21,658 events recorded under the released code’s semantics, and Table 5 decomposes these events by operating mode. The pooled confidence while engaged has mean 0.68 and median 0.80 (the cap value), and the pooled speed-confidence correlation is negative (r = −0.25); this is a composition effect of fetch-pinned low-speed events dominating the engaged set, not a property of the free-mode indicator. The prediction pipeline computes a 0.5 s extrapolation horizon from a three-frame velocity window, gated in the camera node at a 0.2 normalised-units/s minimum speed (virtual-operator input bypasses this gate). Saccade-gated confidence fusion (Section 3.2) lifts the 0.8 confidence cap only after a 400 ms fixation within a 2° angular window; the corresponding gaze-to-motion lead is a modelled 150–300 ms (Figure 8), since the gaze stream ran in simulation. The 10-frame consecutive-frame debounce trades approximately 0.65 s of latency for suppression of transitional misclassifications. A quantitative prediction-error metric (mean Euclidean distance between the extrapolated ghost target and the operator’s eventual hand position at the end of the 0.5 s horizon) would require synchronous ground-truth hand-position logging that was not captured in the single-operator trial run reported here; this metric is identified as a deployment-time logging requirement for the multi-participant evaluation described in Section 6 (Table 5, Part B).

**TABLE 5:** Prediction-pipeline evaluation. Part A: operational statistics measured from the released prediction log (gaze_log.csv), which records one row per hand-velocity event outside torch mode and pools webcam-sourced and virtual-operator-sourced sessions; sessions are concatenated, so proportions are valid but durations are not; the gaze gate operated on the simulated Tobii stream; the first five sessions predate a change to the confidence divisor and are excluded (21,658 events retained). Part B: the endpoint-accuracy metrics requested in review, specified as a defined protocol for the next study; these require logging the predicted endpoint alongside a matched actual-position queue, a software-only instrumentation addition at the prediction publish point.

Evaluation parameter	Definition	Measured value or computation	Baseline/note
Part A (measured): prediction-engagement rate	Share of logged hand-velocity events with non-zero confidence	35.3% (7,640 of 21,658 events)	First five sessions excluded (earlier confidence divisor)
Part A (measured): mode composition of engaged events	Fetch-pinned versus free-following share	67.4% fetch-pinned, capped at 0.8 by the simulated gaze gate; 27.3% free-following (confidence = normalised hand speed, mean 0.32); 1.6% capped with source indistinguishable; 3.6% uncapped	Fetch pinning is a design property, not gaze tracking
Part A (measured): pooled confidence while engaged	Distribution of the displayed indicator	Mean 0.68, median 0.80 (the gaze-gate cap value)	A deliberateness indicator, not an accuracy measure (Section 4.2)
Part A (measured): speed-confidence relation	Pooled correlation between hand speed and confidence	r = −0.25; a composition effect of fetch-pinned low-speed events; within free-following the indicator equals hand speed by construction	Whole-log pooled value
Part A (measured): uncapped engaged events	Events with confidence above 0.99	277 events: 148 during simulated gaze commitment; 129 in two sessions in which the gaze stream was inactive and the fail-open watchdog held the gate open (gaze_speed_dps = 0 throughout)	Watchdog fail-open behaviour disclosed for completeness
Part B (protocol): mean endpoint prediction error	Distance from the predicted reach endpoint to the operator’s actual endpoint	Euclidean distance between the ghost-target position and the final hand position, averaged over completed reaches	Current-hand-position (no-prediction) estimate
Part B (protocol): 0.5 s horizon error	Prediction error at the extrapolation horizon	Mean distance between the predicted position at time t and the actual hand position at t + 0.5 s, over active-prediction frames	Static-hand assumption at time t
Part B (protocol): false pre-positioning rate	Robot pre-positions toward a target the operator does not reach	Wrong-target pre-positioning events divided by total pre-positioning events	Reactive mode (pre-positioning disabled)
Part B (protocol): success rate under direction changes	Predictor recovery after an abrupt reversal of hand direction	Correct endpoint re-acquisitions within a fixed recovery window after a velocity reversal, divided by total reversals	No-prediction baseline

### 4.3 Fatigue adaptation and TEPR

At mild fatigue levels (0.3 ≤ F < 0.6) the motion-interpolation factor decreases from 0.10 to 0.06, producing visibly slower, more deliberate motion without interrupting the fetch sequence. The fatigue estimator fuses four normalised metrics (blink-rate, blink-duration, PERCLOS, hand-jerk) with fixed weights and exponential smoothing, producing a composite score mapped to four bands (FRESH, MILD, MODERATE, SEVERE) that respectively enable full-speed autonomy, reduced speed, OKAY-confirmation-gated motion, and safety lockout. The acute TEPR Soft E-Stop operates orthogonally to the chronic band system and scales both the velocity ceiling and slew cap by 0.1 for a 3-s cooldown.

**FIGURE 9:**
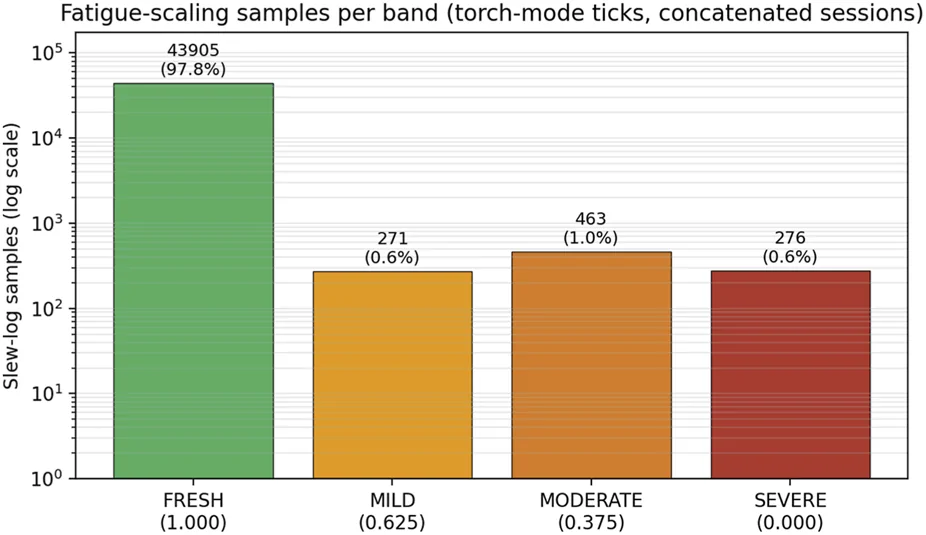
Distribution of the fatigue-dependent velocity-scaling multiplier recorded during free-hand torch-mode operation (slew_log.csv, 44,915 control ticks). The values fall on exactly the four preset band levels (FRESH 1.000, MILD 0.625, MODERATE 0.375, SEVERE 0.000) with no intermediate values, confirming the four-band discretisation. The logger records only torch-mode ticks and excludes all fetch-sequence motion regardless of band (the fetch sequences use a separate trajectory controller that does not write to this log), so the 97.8% FRESH proportion reflects the torch-mode-active subset and a rested operator, not the system’s total operating envelope. Fetch-and-deliver sequences across the fatigue bands, with the fetch controller’s band-aware trajectory-time scaling and SEVERE lockout, are shown on the physical cobot in Supplementary Video S1. Per-tick fetch-mode logging and organic induced-fatigue validation are future work (Section 6). Logarithmic y-axis.

**FIGURE 10:**
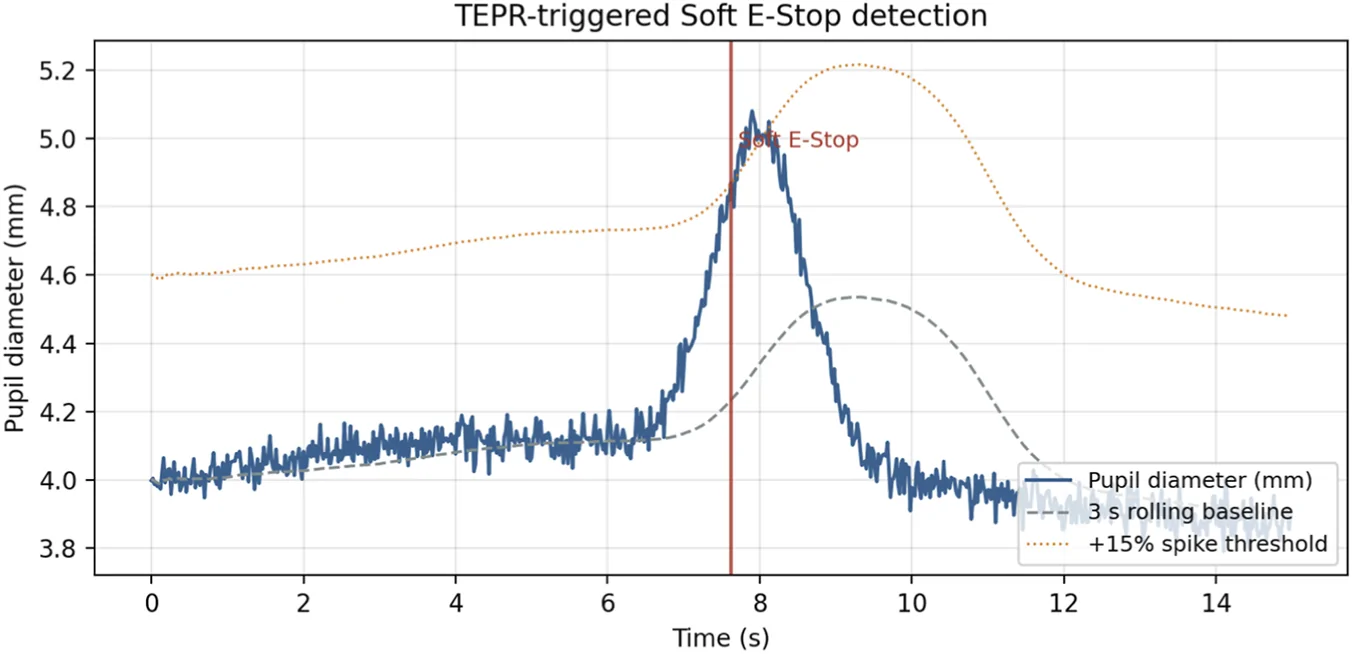
Illustrative TEPR detection timeline generated from a synthetic pupil trace containing a modelled stress dilation, showing the detector geometry: rolling 3-s baseline (dashed grey), +15% spike threshold (dotted orange), and the trigger at the first threshold-crossing sample (red line). This figure is not drawn from the released tepr_log.csv: in that log, recorded under the simulated Tobii stream, no sample exceeded the threshold (maximum excursion 4.8% above baseline) and the trigger column is all zero, because the soft-e-stop episodes reported in this study were exercised through the Wizard-of-Oz channel, which bypasses the pupil comparison (a logged response episode is shown in Figure 11).

**FIGURE 11:**
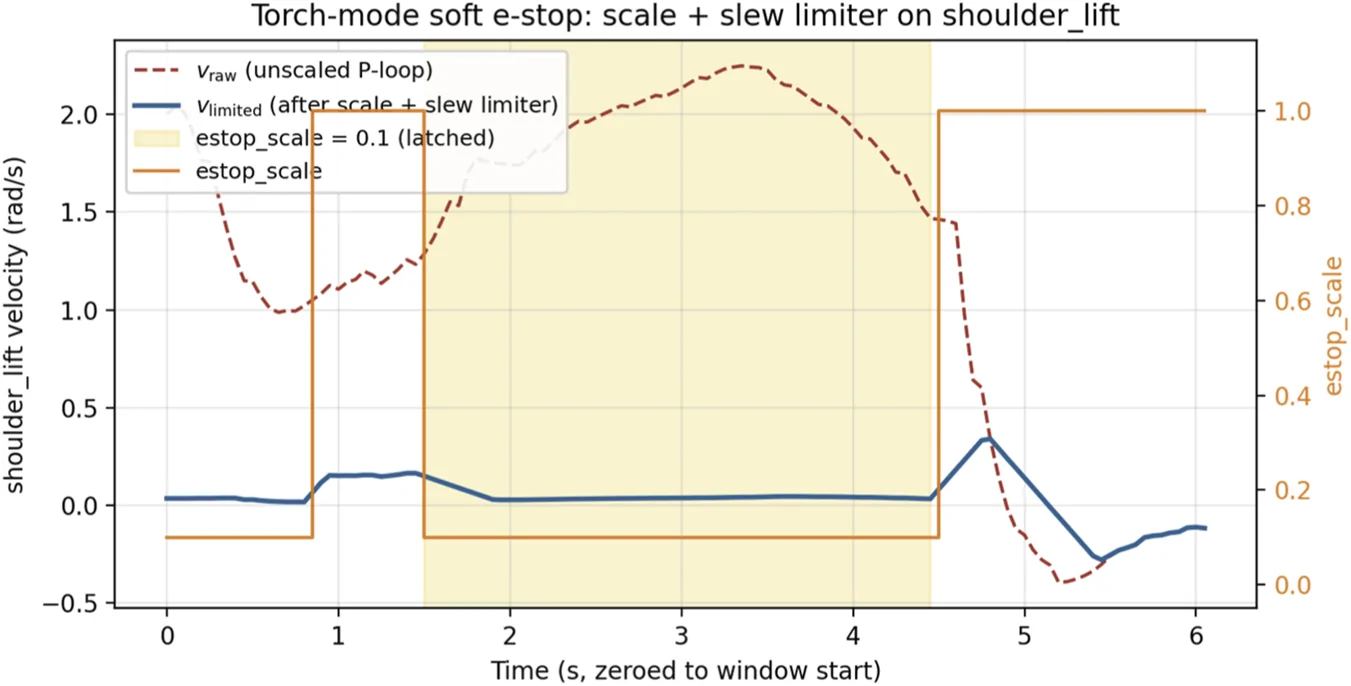
A representative torch-mode soft-e-stop episode on shoulder_lift (slew_log.csv, rows 4,619–4,679; a single contiguous episode within one logged session). Raw P-loop velocity (dashed) climbs to 2.25 rad/s while the limited velocity (solid) is capped at approximately 0.03–0.13 rad/s; estop_scale (orange, right axis) latches at 0.1 during the cooldown window and returns to 1.0 once the cooldown elapses.

Figure 9 confirms the four-band discretisation of the velocity-scaling multiplier: the logged values occupy exactly the four preset levels 1.000, 0.625, 0.375 and 0.000, with no intermediate values, and a logarithmic y-axis is used so all 4 bars remain visible. The slew log that backs this figure records only free-hand torch-mode control ticks and, by construction, excludes all fetch-sequence motion regardless of band, because fetch sequences are executed through a separate trajectory controller that does not write to this log. The 97.8% FRESH proportion therefore reflects the torch-mode-active subset and a rested operator, not the system’s total operating envelope. Fetch-and-deliver sequences were performed across the fatigue bands during the trials, with the fetch controller applying its own band-aware trajectory-time scaling (1.5 times longer at MILD, 2.5 times longer at MODERATE) and a SEVERE lockout; these behaviours are shown on the physical cobot in Supplementary Video S1. Per-tick fetch-mode logging and organic induced-fatigue validation are specified as future work in Section 6. Figure 10 illustrates the TEPR detection geometry on a synthetic pupil trace: the Soft E-Stop fires when the pupil exceeds the 3-s rolling baseline by more than 15%. No threshold crossing occurred in the released tepr_log.csv (maximum excursion 4.8% above baseline), because the soft-e-stop responses reported in this study were triggered through the Wizard-of-Oz channel, which bypasses the pupil comparison; a logged soft-e-stop response episode is shown in Figure 11.

**FIGURE 12:**
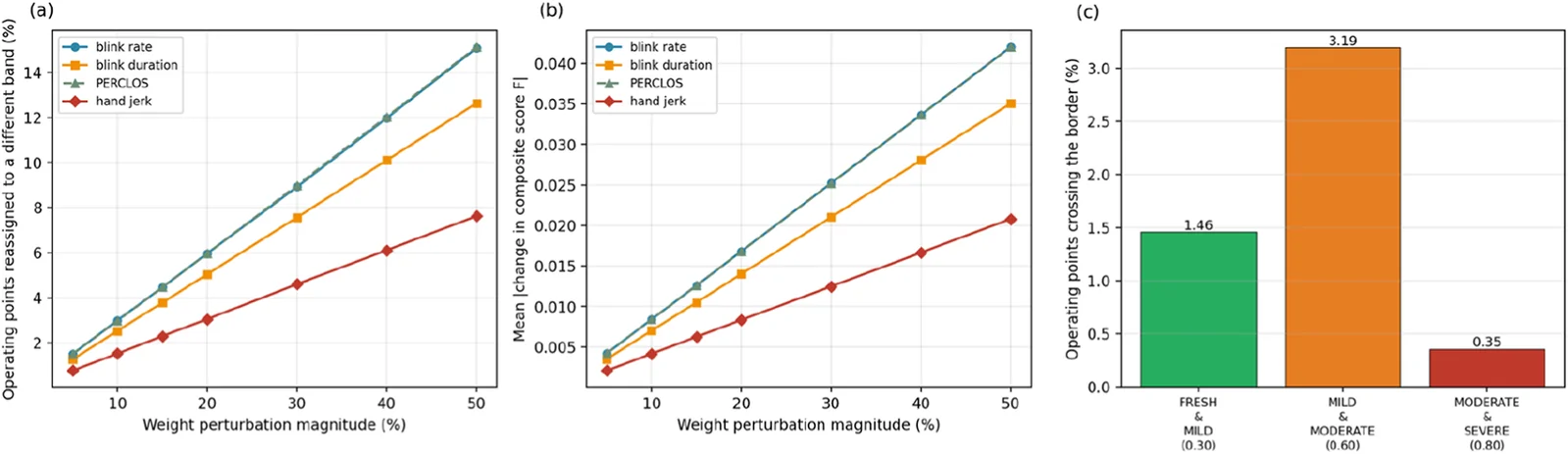
Sensitivity of the band assignment to the composite-score weights, computed analytically across the abstract sub-score domain (uniform sampling, since empirical per-tick sub-score distributions were not logged and the operational data are FRESH-skewed). **(a)** A weight-perturbation sweep reassigns at most about 15% of operating points even at the plus or minus 50% extreme, and only 3%–6% at plus or minus 20%; the blink-rate and PERCLOS curves coincide (equal nominal weight 0.30, PERCLOS drawn dashed for visibility). **(b)** Mean composite-score shift grows smoothly with perturbation. **(c)** The safety-critical MODERATE-to-SEVERE boundary is the most stable (0.35% of points crossing at plus or minus 20%). The perturbation range is a methodological robustness range, not a literature-derived bound.

Because the composite fatigue score is a deterministic weighted sum, the sensitivity of the band assignment to the four weights can be characterised analytically over the abstract sub-score domain, without additional data (Figure 12). Sweeping each weight from a five to a fifty per cent perturbation, while renormalising the remaining weights, reassigns only three to six per cent of operating points to a different band at a plus or minus twenty per cent perturbation, and at most about fifteen per cent at the plus or minus fifty per cent extreme. The safety-critical MODERATE-to-SEVERE boundary is the most stable of the three, with only 0.35 per cent of points crossing it at a plus or minus twenty per cent perturbation, so the weight choice chiefly affects the lower, non-safety-critical bands. The band assignment is therefore robust to the precise weight values.

On the trial day, the Tobii Pro Glasses 3 scene camera displayed SMPTE colour-bar test patterns and the device continued emitting gaze samples with the glasses removed, indicating a hardware fault that reboot and recalibration did not resolve. Trials were therefore run with simulation-mode fatigue (use_sim_tobii = true), exercising the identical downstream control pipeline via Numpad-5 Wizard-of-Oz triggering (Dahlbäck et al., 1993). Organic TEPR-based emergency-stop and organic gaze-commitment were not live-validated on pupil data, but the response mechanisms downstream of each trigger were verified end-to-end through the logged estop_scale and is_fixation columns reported in this section. The plotted episode used torch mode because fetch-phase velocities are conservatively bounded, masking any reduction. Figure 11 shows a 3-s activation on shoulder_lift: estop_scale latches at 0.1 (the fixed 10× target) during the cooldown window and returns to 1.0 once the cooldown elapses, while the slew limiter caps the limited velocity at approximately 0.03–0.13 rad/s as the raw velocity climbs to 2.25 rad/s; the instantaneous limited-to-raw ratio therefore varies between approximately 1.5% and 11.5% across the plotted window, the two mechanisms acting together for a gentle deceleration rather than a hard snap.

### 4.4 State machine behaviour

All state transitions were verified through systematic testing of gesture sequences. POINT correctly initiates robot motion toward the clamped target. STOP immediately freezes the robot regardless of the current state. OKAY correctly unfreezes the robot and confirms pending actions. GRASP commands gripper closure, visible in the RViz simulation. NEUTRAL maintains the current state without triggering actions. Workspace clamping correctly constrains all targets within the defined boundaries, and joint limits prevent any robot link from penetrating the table plane. To make the safety-critical logic auditable, Table 6 documents the finite-state-machine specification (for each state and triggering input, the expected next state) and Table 7 lists the orthogonal safety and fatigue branches that operate across the fetch states. The deployed robot_latency_log.csv captures FROZEN-state activations rather than the full per-state transition graph; observed verification in the present single-operator trials was by live observation of the RViz state display and Gazebo behaviour, and structured per-transition logging for an expected-versus-observed matrix across trials is identified as future work (Section 6).

**TABLE 6:** Fetch-sequence finite-state-machine transitions: the expected next state for each state and triggering input, taken from the controller specification.

Current state	Triggering input/condition	Next state
NONE	POINT lands on a registered object	IDENTIFIED
IDENTIFIED	OKAY (confirm target)	HOMING_TO_FETCH
HOMING_TO_FETCH	Reached pre-grasp pose (joint error < 0.05 rad, dwell > 2 s)	PRE_APPROACHING (Red Cube)/APPROACHING (others)
PRE_APPROACHING	Reached waypoint (joint error < 0.05 rad, dwell > 2 s)	APPROACHING
APPROACHING	Reached grasp pose	WAITING_GRASP
WAITING_GRASP	GRASP gesture	GRASPING
GRASPING	Gripper closed (dwell > 2 s)	RETURNING_HOME
RETURNING_HOME	Reached delivery pose	DELIVERING
DELIVERING	Reached handover pose	WAITING_RECEIVE
WAITING_RECEIVE	OKAY when FRESH or MILD	NONE (release immediately)
WAITING_RECEIVE	OKAY when MODERATE/SEVERE (first press)	WAITING_RECEIVE (hold, await second OKAY)
WAITING_RECEIVE	Second OKAY when fatigued	NONE (release)

**TABLE 7:** Orthogonal safety and fatigue branches that operate across the fetch states.

Branch	Trigger/condition	Effect
STOP freeze	STOP gesture in any state	Enter FROZEN; current fetch state saved; OKAY resumes from the saved state when not SEVERE
SEVERE lockout	Fatigue F ≥ 0.8 with an active POINT command	Enter FATIGUE_LOCKOUT; new POINT-driven commands ignored until fatigue drops below SEVERE
TEPR soft-stop	Pupil-spike trigger (simulated in this study)	Velocity ceiling and slew cap scaled by 0.1 for a 3 s cooldown, then restored
MODERATE gating	Fatigue F ≥ 0.6 in torch mode/at handover	Movement requires an OKAY confirmation (3 s grace window); handover requires a double OKAY

### 4.5 Real-time performance and system-level latency

The end-to-end pipeline is designed to run deterministically on commodity hardware (Intel i7, no GPU required). Measured per-stage periods are: MediaPipe hand-landmark extraction at an effective rate of approximately 15 Hz (a 30 Hz timer limited by full-HD inference, Figure 6), forward-velocity control loop at 20 Hz, and RViz marker publishing at 2 Hz. The worst-case operator-gesture-to-robot-motion latency comprises three terms: up to one camera-driver capture-buffer interval, bounded above by approximately 130 ms for the default four-frame V4L2 buffer at the nominal 30 Hz capture rate; the 10-frame consecutive-frame debounce, which at the measured approximately 15 Hz effective inference rate spans approximately 650 ms and dominates the budget; and one 50 ms forward-velocity control tick. The conservative end-to-end ceiling is therefore approximately 830 ms. The safety-relevant STOP gesture uses a 2-frame fast-debounce branch and is therefore substantially faster than the full-gesture path. The saccade-gated early-commit pathway (Section 3.2) is designed to recover a further modelled 150–300 ms, and the TEPR Soft E-Stop operates on an independent 300–500 ms channel. These figures sit below the 1.0 s response-time limit at which an interactive system begins to interrupt the user’s flow of thought (Nielsen, 1993), the general usability guideline adopted here as the design target for perception-to-actuation latency. Figure 6 shows the inter-sample gesture-classification latency per class from latency_log.csv. Median latency is 64–65 ms across all five classes with 95th-percentile values of 69–77 ms, consistent with the approximately 15 Hz MediaPipe inference rate and the consecutive-frame debounce described in Section 3.1. The deterministic TOGGLE detector is excluded because it is a deterministic override rather than a Random-Forest class.

## 5 Discussion

### 5.1 Significance of predictive behaviour

The velocity-based intention prediction, simpler than affordance-based approaches (Koppula and Saxena, 2016) or trajectory-planning methods (Mainprice and Berenson, 2013), is designed to support anticipatory pre-positioning; whether this yields a measurable improvement in responsiveness or fluency is deferred to the evaluation protocol of Section 6 (Table 5, Part B). The 0.5 s horizon aligns with the duration of a pointing transition, so the robot is intended to begin moving toward the new target approximately when the operator’s hand decelerates at the destination. Linear extrapolation assumes constant velocity over the horizon and cannot anticipate direction changes. For the dominant use case of sequential pointing this is computationally efficient; richer prediction models are left for future work on trajectories with complex curvature.

### 5.2 Closing the HCDT loop

The principal contribution of this work is the closing of the feedback loop in the Asad et al. (2025) three-module HCDT framework. Their framework provides the predictive outputs required for a complete HCDT and scopes the implementation of feedback and physical-assistance mechanisms as the next stage of the framework’s development. The present paper supplies that feedback layer in a form designed for real-time operation on standard cobotic hardware, without sacrificing the modular substitution path that makes the Asad et al. architecture valuable in the first place. Module realisations differ deliberately from those in Asad et al. (2025): a Vision-Language Model can describe a kitchen-assembly task in natural language, but an FSM can be safety-reviewed line by line; a diffusion-based motion model can synthesise plausible novel motions, but an analytical IK solver delivers sub-millisecond joint solutions on CPU. Because the architectural skeleton is preserved, the two instantiations are complementary rather than competing.

A separate closely-related contribution by Escobar-Naranjo et al. (2026), published in the same Research Topic in May 2026, proposes a multimodal-intention HRC framework with a CNN-LSTM-Transformer cognitive model and a Soft Actor-Critic deep-reinforcement-learning controller on a uFactory Lite 6 cobot. The two papers are structurally complementary rather than competing, for three reasons. First, the article types differ: Escobar-Naranjo et al. publish their work as Hypothesis and Theory, with empirical validation explicitly out of scope (“at the time of writing, this protocol has not yet been executed”), whereas the present paper publishes as Original Research and reports measured 99.62% classifier accuracy, measured 64–65 ms median classification latency on the same step as their projected 80–100 ms control loop (Figure 6), a conservative end-to-end gesture-to-motion worst-case ceiling of approximately 830 ms, and a deployed ten-state finite-state machine running on the physical UR3. Second, both papers extend Asad et al. (2025) (Escobar-Naranjo et al. (2026) adopt the same gaze-attention formulation), but the signal stacks differ: their pipeline fuses RGB-D, gaze and wrist-mounted force/torque, while the present work fuses RGB-camera landmarks with wearable Tobii Pro Glasses 3 pupillometry and adds a composite fatigue score F and a Task-Evoked Pupillary Response soft emergency stop, neither of which appear in their formulation. Third, the cognitive layer differs in interpretability: Escobar-Naranjo et al. (2026) acknowledge in their §4.1.4 that their deep-learning and DRL components are inherently black boxes and that this poses a challenge for operator trust and safety certification, whereas the Random Forest classifier with an explicit geometric override layer used here is auditable line by line, which is the property exploited by the override layer described in Section 3.1. The two contributions are therefore best read in parallel: theirs as a formally grounded methodological proposal awaiting empirical study, the present paper as a deployed empirical instantiation on a different cobot platform with a complementary signal stack.

### 5.3 Fatigue monitoring considerations

The integration of fatigue monitoring with robot control remains relatively unexplored in HRC research (Carissoli et al., 2024); the graduated four-band response (speed reduction, confirmation gating, lockout) addresses this gap. The Tobii Pro Glasses 3 provide more reliable PERCLOS and pupil measurements than camera-based alternatives, though at the cost of wearable instrumentation.

Recent reviews of digital-twin applications across human factors and ergonomics confirm that closed-loop physiological feedback remains uncommon in practice (De Angelis et al., 2025), underlining the contribution of the four-band response. Beyond its primary safety function, the visible speed reduction also acts as a trust-calibration signal to the operator: under the human-factors framework of Pietrantoni et al. (2024), an observable reduction in robot velocity communicates that the system is aware of operator state, helping the operator neither over-trust nor under-trust the cobot and complementing the safety bound with a continuous ambient feedback channel.

### 5.4 Relevance to safety standards

The STOP-gesture override and the workspace-clamping system are research-layer safety behaviours inspired by the safety-rated monitored stop and speed-and-separation monitoring of ISO 15066:2016, but they are not certified safety functions: they are implemented on non-safety-rated commodity sensing (an RGB webcam) and a non-safety-rated control chain, so they do not provide the guarantees those standards require. Their value here is to show that fatigue-adaptive behaviour can extend collaborative-safety thinking into the operator’s psychophysiological state, a dimension not currently addressed by the standards. A certified industrial deployment would require safety-rated sensing and control hardware, a certified emergency-stop interface, a formal task-based risk assessment to ISO 12100, and redundancy and diagnostic coverage for the stop function; these are outside the scope of the present research prototype.

## 6 Limitations and future work

Several limitations are acknowledged. The gesture system, reliable for the five defined classes, does not handle continuous or parameterised gestures. Fatigue thresholds are literature-derived rather than per-operator calibrated, and velocity prediction assumes linear trajectories. The evaluation reflects a single-operator session; multi-participant studies remain necessary to validate the predictive-and-adaptive behaviour at scale. A single-subject dataset does not provide statistical grounds for claims of generalisation across operators, anthropometrics, lighting or behavioural variation; the reported gesture accuracy characterises within-session performance rather than cross-user performance, and cross-user validation is required before any such claim. On the trial day the Tobii Pro Glasses 3 displayed a hardware fault, and trials were therefore run with simulation-mode fatigue and Wizard-of-Oz triggering of the TEPR pathway; the downstream response mechanisms were verified end-to-end through logged signals, but organic TEPR-based emergency-stop and organic gaze-commitment were not live-validated on pupil data.

Future work will focus on organic, hardware-in-the-loop validation of the TEPR and gaze-commitment pathways on functioning eye-tracking hardware, formal multi-participant studies of task time and subjective workload, and personalised fatigue-threshold calibration through a short baseline-recording phase at session start. Cartesian impedance control is a further natural extension: the current velocity-controlled pipeline handles operator-state-driven adaptive speed but does not yield compliantly to contact forces, which would be required for force-modulated tasks such as polishing, deburring or hand-guided teaching. The most significant forward direction is integration of the three-module pipeline into a full Human-Centric Digital Twin (Asad et al., 2023): coupling the reasoning module to a VR/AR virtual twin for remote supervision and training, lifting the fixed gesture vocabulary into a Vision-Language-Model task graph (Asad et al., 2025), and closing the loop so the cobot anticipates rather than merely reacts.

To place these directions on a rigorous empirical footing, a defined evaluation protocol is specified for the next study, following the within-subjects design now standard for proactive-HRC evaluation. At least thirty participants (inclusion criteria: normal or corrected vision, no prior human-robot-collaboration experience) would each experience the system under counterbalanced conditions, a reactive baseline and the predictive, fatigue-aware mode, presented in a Latin-square order to control for learning and fatigue effects. Objective measures would include task completion time, operator and robot idle time, the number of safety stops, end-effector jerk, and end-to-end gesture-to-motion latency measured directly from a timestamped gesture-onset stimulus to the first robot-motion response, rather than reconstructed from a component budget. Gesture accuracy, geometric-override behaviour, fatigue-band exposure and end-to-end latency would additionally be evaluated across varied lighting conditions and camera configurations, beyond the single overhead setup used here. The prediction module would be evaluated with mean endpoint prediction error, 0.5 s horizon error, false pre-positioning rate, and success rate under direction changes, each compared against a no-prediction baseline; Table 5 reports the operational statistics already measurable from the released logs (Part A) and consolidates each requested metric, its computation and its baseline (Part B). Subjective workload and trust would be captured with the NASA-TLX and standardised Likert instruments. Fatigue-band transitions would be exercised with an induced or prolonged-fatigue protocol so that the bands are entered on organic pupillometry rather than injected triggers, and the composite-score weights and thresholds would be calibrated per operator through a defined protocol: a rested-baseline window of the order of sixty to 90 seconds is recorded at session start while the operator is alert, capturing resting blink rate, blink duration, PERCLOS and pupil diameter; these values are stored as the operator’s personal baseline; each fatigue sub-score is then normalised against that baseline rather than against fixed literature values, so fatigue is measured as deviation from the operator’s own rested state; the four band thresholds are set relative to the per-operator distribution rather than as fixed cut-offs; and, once labelled fatigue data have been collected per operator, the four composite weights themselves are tuned per operator. Differences across conditions would be assessed with repeated-measures ANOVA, or the Friedman test for ordinal data, with Bonferroni correction at a significance level of 0.05. Institutional ethics approval covering human participants would be obtained before data collection.

## 7 Conclusion

This paper has presented the design, implementation and evaluation of a predictive, fatigue-aware human-robot collaboration system for the Universal Robots UR3, integrating non-contact vision-based sensing, gesture classification, velocity-based intention prediction and physiological fatigue monitoring within a modular ROS 2 architecture. The key achievements are: (i) real-time recognition of five gesture classes at 99.62% accuracy on a leak-free split-then-augment evaluation, with both errors falling in the Okay-Neutral pair that the geometric override layer is designed to catch at the system level (cross-session and cross-operator quantification of the override-layer effect is scoped for the multi-participant evaluation described in Section 6); (ii) velocity-based intention prediction enabling anticipatory pre-positioning; (iii) a composite fatigue score derived from eye-tracking and hand-movement metrics; and (iv) an adaptive control architecture modulating robot behaviour according to operator state. The complete software stack is released open-source. To the authors’ knowledge, this is the first such release coupling vision-based intent recognition with physiological fatigue and acute-stress adaptation on a standard collaborative robot, and offers a concrete step toward the human-centric, resilient cobotic systems envisaged by Industry 5.0.

## Data Availability

The complete software stack, training data, trained classifier, URDF-xacro models, the trial logs analysed in Section 4 and the sensitivity-analysis outputs (sensitivity_results.csv), and reproducibility instructions are released under the MIT licence at https://github.com/RaniduGoonetilleke/Predictive-Fatigue-Aware-Human-Robot-Collaboration. A demonstration of the system on the physical cobot (Supplementary Video S1) is also available at https://www.youtube.com/watch?v=RqbxJ8OQCOU.

## Supplementary material

Supplementary Video S1 Demonstration of the deployed system on the physical UR3: fetch-and-deliver across the fatigue bands, STOP/OKAY recovery, torch mode and hazard-zone denial.
