# Correction: Predictive fatigue-aware human-robot collaboration: a real-time, open-source ROS 2 instantiation of a three-module human-centric digital twin framework on a standard cobot

**DOI:** 10.3389/frobt.2026.1964098

**Published:** 2026-09-03

**Authors:** Ranidu P. Goonetilleke, Azfar Khalid

**Affiliations:** Digital Innovation Research Group, Department of Engineering, School of Science and Technology, Nottingham Trent University, Nottingham, United Kingdom

**Keywords:** gesture recognition, human-centric digital twin, human-robot collaboration, Industry 5.0, intention prediction, operator fatigue, ROS 2, TEPR

The statement that financial support was received for this work was erroneously included. The support provided by the Digital Innovation Research Group, Department of Engineering, School of Science and Technology, Nottingham Trent University was not financial in nature. The correct Funding statement appears below.

“The author(s) declare that financial support was not received for the research and/or publication of this article.”

In the acknowledgements, the resources provided by the Digital Innovation Research Group were omitted. The corrected Acknowledgements statement appears below.

“The authors thank the technical staff of the Department of Engineering at Nottingham Trent University for laboratory support during system development. This work was carried out using resources of the Digital Innovation Research Group, Department of Engineering, School of Science and Technology, Nottingham Trent University.”

The original version of this article has been updated.

